# The influence of a large build area on the microstructure and mechanical properties of PBF-LB Ti-6Al-4 V alloy

**DOI:** 10.1007/s00170-022-10671-9

**Published:** 2023-01-06

**Authors:** Axieh Joy I. Bagasol, Frederico R. Kaschel, Saranarayanan Ramachandran, Wajira Mirihanage, David J. Browne, Denis P. Dowling

**Affiliations:** 1 School of Mechanical & Materials Engineering, University College Dublin, Belfield, Dublin 4, Ireland; 2 Department of Materials, The University of Manchester, Sackville Street Building, Manchester, M1 3BB UK

**Keywords:** PBF-LB, Ti-6Al-4 V, Part placement, Microstructure homogeneity, HIP

## Abstract

This study investigated the print homogeneity of Ti-6Al-4 V alloy parts, when printed over a large build area of 250 $\times$ 250 $\times$ 170 mm^3^, using a production scale laser powder bed additive manufacturing system. The effect of part location across this large build area was investigated based on printed part porosity, microstructure, hardness, and tensile properties. In addition, a Hot Isostatic Pressing (HIP) treatment was carried out on the as-built parts, to evaluate its impact on the material properties. A small increase in part porosity from 0.01 to 0.09%, was observed with increasing distance from the argon gas flow inlet, which was located on one side of the build plate, during printing. This effect, which was found to be independent of height from the build plate, is likely to be associated with enhanced levels of condensate or spatter residue, being deposited at distances, further from the gas flow. Despite small differences in porosity, no significant differences were obtained for microstructural features such as prior* β* grain, $\alpha$ lath thickness, and phase fraction, over the entire build area. Due to this, mechanical performances such as hardness and tensile strengths were also found to be homogenous across the build area. Additionally, it was also observed based on the lattice constants that partial in-situ decomposition of ${\alpha }^{^{\prime}}\to \alpha +\beta$ phases occurred during printing. Post HIP treatment result showed a decrease of 7 and 6%, in the yield strength (YS) and ultimate tensile strength (UTS), respectively, which was associated with a coarsening of $\alpha$ lath widths. The potential of the laser powder bed system for large area printing was successfully demonstrated based on the homogenous microstructure and mechanical properties of the Ti-6Al-4 V alloy parts.

## Introduction

Additive manufacturing (AM) has gained considerable interest in the aeronautical, biomedical, and automobile industries for the production of highly complex three-dimensional (3-D) components, which would be difficult to fabricate using conventional manufacturing techniques [1]. In addition, other benefits of AM include a significant reduction in material usage and lead processing time, which can result in cost savings for small batch runs [2]. Whilst there are a range of processing technologies used for the AM of metallic components, one of the most widely applied technologies commercially is the Powder Bed Fusion (PBF) technique. The selective laser melting (SLM) technology is a type of PBF technique, which uses a laser beam (LB) energy source to selectively melt metallic powder particles [3]. During the printing process, a layer-by-layer deposition strategy is used, where the powder layer is exposed to the laser source, melting the powder according to a pre-determined toolpath. The deposition takes place within an inert chamber filled with a continuous flow of argon (Ar), or helium (He) gas, to protect what in many cases are the highly reactive metallic powders.

Ti-6Al-4 V which is the focus of this paper, is an example of an alloy that has been widely used in AM, particularly in the biomedical industry due to its combination of unique properties, including excellent high specific strength, corrosion resistance and biocompatibility [4]. Typically, the microstructure of Ti-6Al-4 V obtained by the LB system consists of extremely fine, non-equilibrium acicular ${\alpha }^{^{\prime}}$ martensite embedded within the columnar prior $\beta$ grain boundaries [5]. It is interesting to note that this microstructure is considerably different from that obtained using conventional manufacturing techniques such as forging, due to the different thermal history during processing [6]. In the case of PBF-LB fabricated Ti-6Al-4 V, the martensitic ${\alpha }^{^{\prime}}$ is formed by diffusion-less transformation due to the rapid heating and cooling rates (~ 10^3^–10^8^ °C/s), experienced during the printing process [7]. Qian et al. [8] reported the maximum cooling rate in the melt pool of Ti-6Al-4 V ranges from 1.2 to 4.0 $\times$ 10^4^ °C/s. Feature sizes such as ${\alpha }^{^{\prime}}$ lath and prior *β* grain width can vary significantly between samples due to thermal cycling; Kaschel et al. [4] measured the *β* columnar grains width between 110 and 200 m$\mu$ and [9] between 100 and 300 $\mu$m. The ${\alpha }^{^{\prime}}$ laths, on the other hand, can vary between 0.3 $\mu$m [5] and 0.6–2.2 $\mu$m thick [4]. More importantly, Sharma et al. [10] reported that the changes in the ${\alpha }^{^{\prime}}$ lath width significantly affect the tensile properties of the components. In addition, the steep thermal gradient results in a significant build-up of thermal residual stresses, which has also been reported by Xu et al. [5] and Kaschel et al. [11] to result in high strength with poor ductility (< 9% elongation).

PBF-LB processing is complex, with a range of parameters influencing the microstructural properties of the printed alloy parts. Examples of these are energy density [12], scanning parameters [13], powder bed temperature [14], build height [7, 15], and build orientation [9]. Moreover, processing parameters must be optimised to avoid the formation of porosity and cracks, which have been reported by Leuders et al. [16], to greatly influence the properties of the fabricated components. These pores act as strong stress raisers and lead to failure, especially under fatigue loading [17]. Post-process thermal treatments are routinely carried out to enhance the mechanical properties of the printed parts. One of the most widely used post-heat treatment techniques applied to PBF-LB parts is Hot Isostatic Pressing (HIP). The method is carried on chemically clean components in a heated, argon-filled vessel, at pressures ranging from 69 to 103 MPa and temperatures between 900 and 955 °C [6]. This treatment technique has been shown to significantly reduce internal porosity, induce stress relaxation, and decompose the ${\alpha }^{^{\prime}}$ martensitic phase into equilibrium $\alpha + \beta$ phase in Ti-6Al-4 V, resulting in superior mechanical properties [9].

**Fig. 1:**
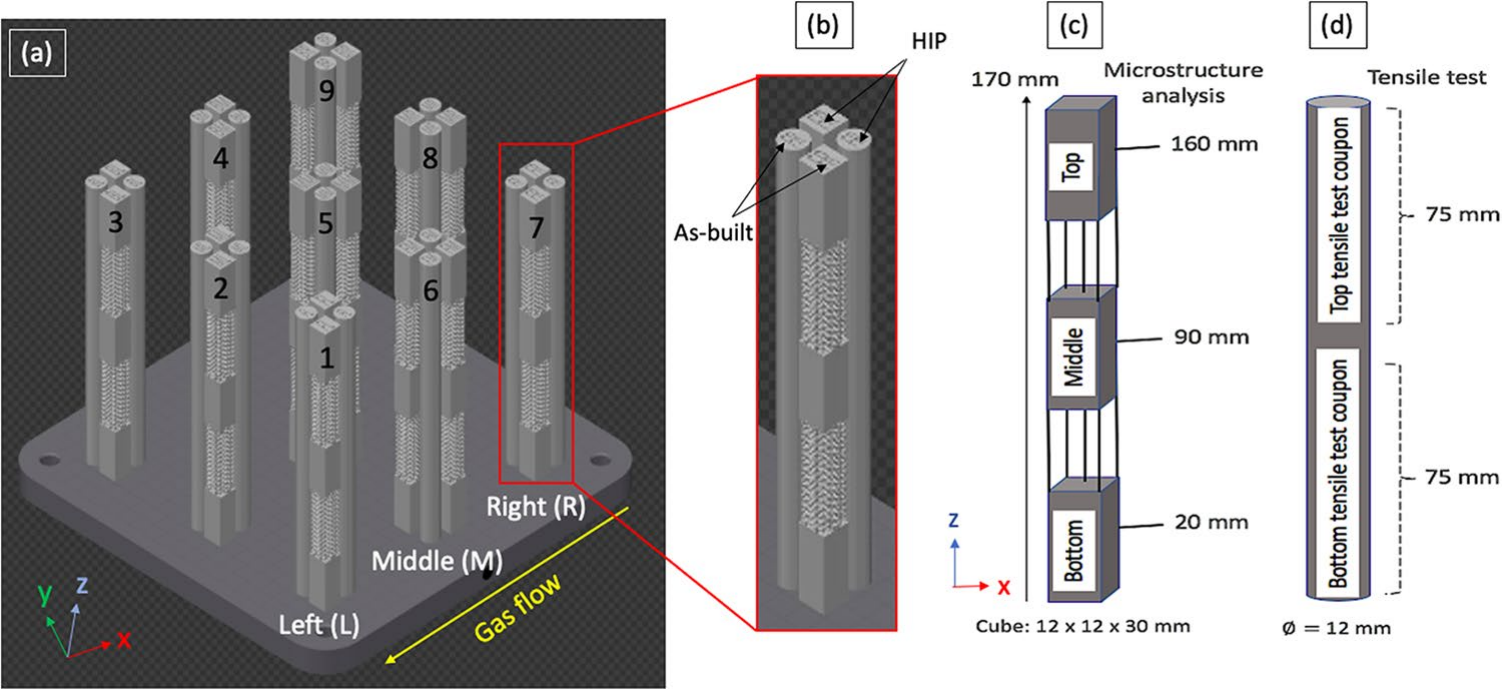
Schematic representation of the PBF-LB build area showing the sample clusters located in Left (1–3), Middle (4–6), and Right (7–9) (**a**). For each cluster, these were divided equally to evaluate the as-built and HIP-treated samples (**b**). Six rectangular coupons (**c**) and two test cylinders (**d**) were available at each location for as-built and HIP. Just note that in all cases, the bottom and top of the print were tested

The focus of the current study is to investigate the influence of the build area on the mechanical and material properties of the printed Ti-6Al-4 V alloy. There have been several reports in the literature on the influence of build area on printed alloy properties obtained using electron beam systems (PBF-EB). For example, Sharma et al. [10] investigated the properties of Ti-6Al-4 V alloy on a 190 × 100 × 120 mm^3^ sample area. However, there have been relatively few reports related to PBF-LB. As demonstrated in Fig. 1, very few considered the impact of Z direction, and the impact of large build was not considered.

As shown in Table 1, Gangireddy et al. [18] investigated the influence of part placement on the build plate (XY-plane) on the dynamic compression behaviour of Ti-6Al-4 V alloy. It was reported that the properties of parts printed at five different locations (four corners and centre) across the build plate displayed heterogeneous microstructures, as well as significant internal stresses, which contributed to the variation in results obtained. Pal et al. [19] reported that location on the build plate (start line and end line of powder spreading) results in increased porosity. This was reported to be due to a variation of powder particles compaction and packing densities during powder spreading of Co-Cr-W-Mo alloy. The paper by Lui et al. [7] is an example of a study which investigated the influence of build height on the properties of printed Ti-6Al-4 V alloy parts, up to 75 mm in height. Variations in both the $\alpha$ lath thickness and grain morphology were observed with height. In contrast for the taller PBF-LB build of 13 × 20 × 114.5 mm, obtained with 316L stainless steel, no significant difference in microstructure was reported with height [15]. The authors reported however that porosity, melt pool geometry, and hardness were directly influenced by build height.

**Table 1:** Examples of build area reported in the literature for various materials printed on the PBF-LB system

References	Material	XY-plane (mm)	Z-plane (mm)
Lui et al. [7]	Ti-6Al-4 V	12 × 12	75
Mohr et al. [15]	316L SS	13 × 20	114.5
Pal et al. [19]	Co-Cr-W-Mo	90 × 90	8
Gangireddy et al. [18]	Ti-6Al-4 V	250 × 250	5

This study investigates, for the first time, the effect of large build area printing using a production scale PBF-LB system, on the properties with location, on the resulting AM alloy parts. The investigation is carried out for Ti-6Al-4 V alloy over a build area of 250 × 250 × 170 mm^3^. In addition, the effect of a post-thermal HIP treatment on these parts is also evaluated and compared with the as-built components.

## Materials and methods

### Sample preparation

Extra-low interstitial (ELI), Grade 23 Ti-6Al-4 V alloy powders, complying with ASTM B348-19, and with particles sizes in the range 21–48 $\mu$m were used for the printing studies, which were carried out using the Renishaw 500S AM production scale system. This system is equipped with a 500 W Yb:YAG (*λ* = 1080 nm) single laser, which follows a Gaussian beam profile, achieved using a dynamic focusing system that uses two galvo mirrors, correcting the near-parabolic focal length change required as the beams travel across the bed, maintaining the beams in focus. In this study, the build parameters were kept constant throughout the printing process, over the 250 × 250 × 170 mm^3^ build area investigated. Table 2 details several key processing parameters used in the manufacturing of the samples. The overall volumetric energy density (VED) used to print the samples was 50 J/mm^3^ calculated using the energy equation reported in [20].

**Table 2:** PBF-LB parameters used in coupon fabrication of test specimens

Laser power (W)	Hatch distance (μm)	Hatch offset (μm)	Layer thickness (μm)	Exposure time (μs)	Rotation increment angle (°)	Point distance (μm)
400	100	50	60	60	67	80

As the objective of this study is to investigate the influence of part placement in the XYZ positions, nine 30 mm sized clusters of test prints were evenly placed across the 250 × 250 mm build area, as shown in Fig. 1. The evaluation of print homogeneity in the Z-plane was carried out by printing three rectangular coupons (12 × 12 × 30 mm^3^), with a build height of 170 mm. The influence of build height was evaluated at 20 mm (B—bottom), 90 mm (M—middle), and 160 mm (T—top), where the mid-point of the test samples was prepared and subjected to microstructural examination. For Electron Back Scattered Diffraction (EBSD), the samples were sectioned from the mid-sections of the broken tensile test samples.

Adjacent to the microstructure test coupons were cylindrical coupons ($\varnothing$ 12 × 170 mm^2^), from which two 75 mm tensile coupons were machined, according to ASTM E8-16a. The locations in the XY-plane were abbreviated as follows: ‘*Left*’, ‘*Middle*’, and ‘*Right*’ as shown in Fig. 1(a). This figure illustrates the three clusters of samples located on the ‘*Right (1–3)*’, ‘*Middle (4–6)*’, and ‘*Left (7–9)*’ of the build plate, positioned at approximately 10, 140, and 235 mm from the front-edge of the plate, respectively. These test samples were used for alloy tensile and microstructure property evaluation, as shown schematically in Fig. 1c, d. Overall, 54 rectangular coupons and 18 cylindrical samples were printed. The test specimens were directly printed onto the plate, which had been preheated at 170 °C. After printing, the samples were removed from the plate using wire electro-discharge machining (EDM). In addition to the as-built condition, some of the printed test samples were used to evaluate the effect of a post-print hot isostatic pressed (HIP) treatment. The latter involving treatment at 100 MPa and 920 °C for 2 h, in an inert atmosphere [21].

For microstructural characterisation studies, samples were mounted in Bakelite, to facilitate grinding using SiC papers with grit size in the range 180 to 2500. The parts were then polished using a 9 $\mu$m diamond suspension, followed by a 0.04 m$\mu$ OPS colloidal silica solution. To facilitate the examination of the microstructural features, etching was carried out using Kroll’s reagent (2% HF, 5% HNO_3_ and 92% deionised water), for 15 s. For EBSD, all the samples were finally polished with a 0.04 μm colloidal silica solution for a long duration in addition to the standard grinding (400, 800, 1200, and 2400 SiC grit) and polishing routes (6 µm, 3 µm, and 1 µm). As the freshly prepared sample surface had some subsequent oxidisation in ambient environmental conditions, the final polished samples were immediately examined in an SEM to perform the EBSD analysis. EBSD was performed using a Field Emission Scanning Electron Microscope (FE-SEM, Tescan Mira3) at 20 kV to measure the grain size as well as their relative orientations. For the EBSD measurements, the samples were tilted at 70° to collect the diffracted electrons emitted from the crystal lattices and to record these diffraction patterns through the EBSD detector (Symmetry Detector, Oxford Instruments). EBSD scans were performed using a step size of 0.2 µm to achieve the best spatial resolution during the EBSD mapping. All the EBSD scanned datasets were post-processed by the AZtec software for further quantitative microstructural analysis such as grain misorientation distributions, grain size statistics, and phase analysis.

### Characterisation of the Ti-6Al-4 V alloy

#### Porosity measurements

X-ray computed tomography (CT) measurements were conducted using GE Phoenix Nanotom system, operating at a 150 kV and 200 $\mu$ A. This system has a resolution of approximately 7 $\mu$m. The measurements were taken from the gauge area (12 mm $\times$ $\mathrm{\varnothing }$ 6 mm), of the tensile specimens printed at each position. A total of 3 bottom and 3 top samples were subjected to CT scanning. Each scan lasted approximately 9 min, and a total of 1079 ‘slice’ images were obtained. The resolution of the scans was set at 7 $\mu$m, defined as the smallest detectable pore size in the samples. Post-processing analysis of the raw CT data was carried out to facilitate porosity measurements, using the Porosity/Inclusion analysis add-on module on VG Studio Max 3.4 [22]. Within this module, VGDefX defect detection was used for an optimised calculation of defects.

#### Microstructure measurements

Phase identification measurements were carried out using the Siemens D500 X-ray Diffractometer (XRD), operated at 40 kV and 30 mA with a Cu Kα radiation (*λ* = 0.1540). Acquisition for phase identification was carried out using a *2θ* range between 30 and 90°, with a dwell time of 1.5 s and step size of 0.04°. Indexing of the XRD patterns was carried out using Crystallography Open Database (COD) according to the XRD patterns of Ti-6Al-4 V alloy reported by [23]. The XRD data was processed using OriginPro software. Equation 1 was used to account for the instrumental broadening using Si standard reflection, B_i_ (0.0013 rad), when analysing the full-width at half maximum (FWHM) before strain analysis using Williamson-Hall method [24]. The microstructure was analysed using Olympus Gx51 Inverted Optical Microscope and Hitachi 4000 Scanning Electron Microscope (SEM) at 15 kV using back-scatter electrons (BSE) detector. Quantification of both the *β* grain and $\alpha$ lath widths was carried out based on the approach taken by [25]. Three images were obtained for each sample and each of these images; 15 $\alpha$ laths were measured (a total of 45 measurements).

1 $${B}_{r}=\sqrt{{b}_{2}-{b}_{2i}}$$

#### Mechanical testing

Vicker’s hardness testing was performed using a Leitz Microhardness Tester, with an applied load of 500 g (HV 0.5) for a period of 10 s, following the method set out by the International Standard ISO6507-1. For each sample, a minimum of 10 measurements were taken from the centre region, away from the contour melt area. Two dog bone tensile specimens (from bottom and top) were machined from a single rod as shown in Fig. 1, conforming to ASTM E8-16a, specimen 3 (6 mm diameter and 24 mm gauge length), using computer numerical control (CNC) turning. Samples were tested using a Tinius Olsen 50kN Benchtop Tester equipped with a clip-on extensometer according to BS EN ISO 6892–1. Samples were subsequently subjected to fractography analysis.

## Results

The objective of this study is to investigate the influence of the build area on the porosity, micro-structure, micro-strain, and mechanical properties (hardness and tensile strengths), of the as-built and post-print HIP-treated Ti-6Al-4 V alloy parts. The following details the results of each of these investigations.

### Porosity characterisation

**Fig. 2:**
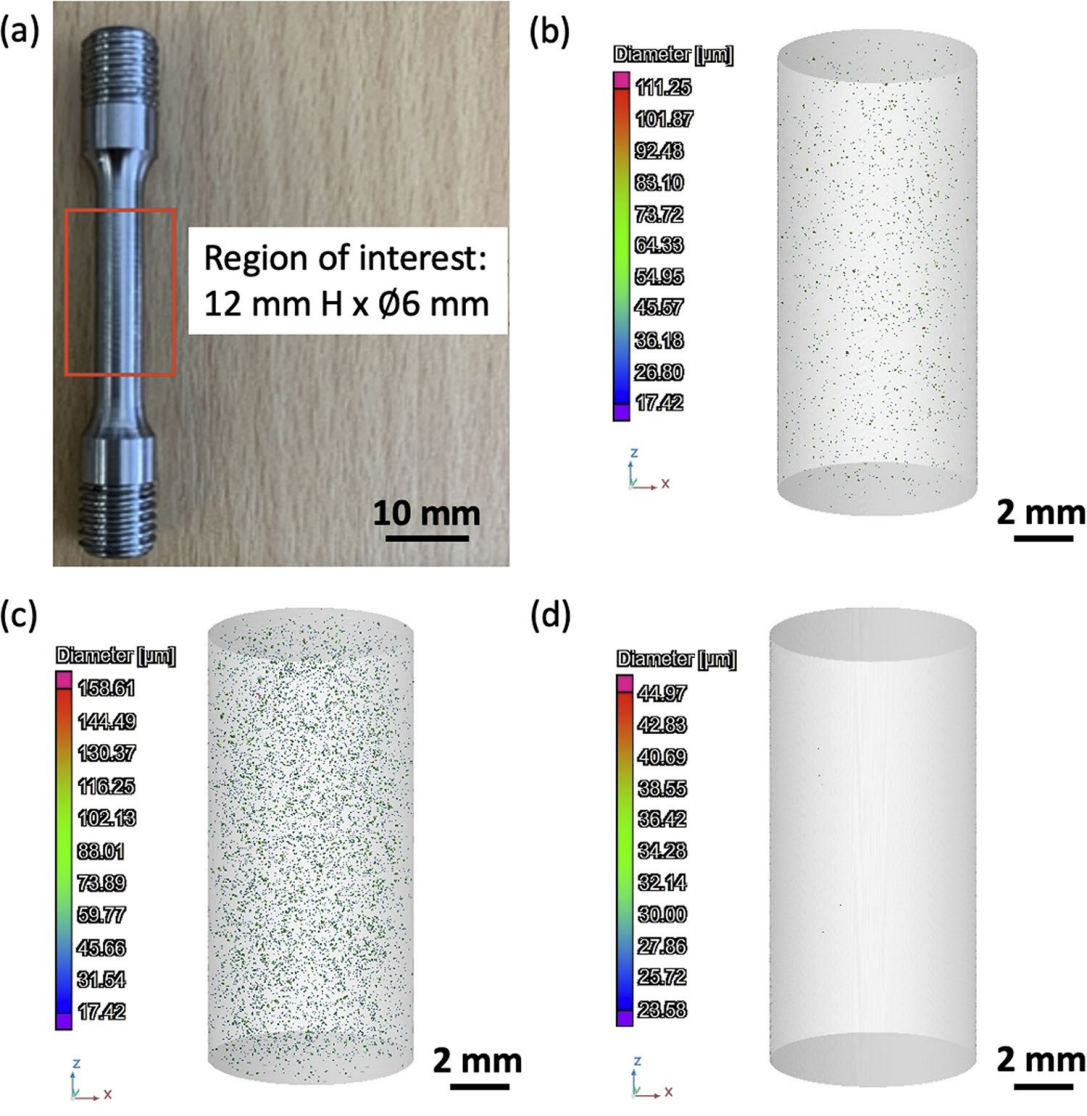
Region of interest in the tensile gauge Sect. (12 mm $\times$ $\varnothing$ 6 mm) of as-built sample (**a**), CT image of a part printed adjacent to the gas outlet (**b**), and printed away from the gas outlet (**c**). Low porosity was determined using VGDefX analysis after HIP treatment (**d**). Most defects found in this region were spherical voids largely associated with gas porosity, with diameters of less than 160 $\mu m$

CT analysis of parts fabricated at different XYZ positions facilitated the measurement of defect volume, pore size, and geometry, with build location. Figure 2 illustrates the pore distribution within the gauge area of the tensile test specimens. Based on porosity measurements, the porosity defect volumes obtained were 0.01% (right), 0.04% (middle), and 0.09% (left), across the build plate in the XY-plane (see Table 3), whilst for the Z-plane results indicate that there is a negligible difference in porosity between samples. This is in contrast to the results obtained by Mohr et al. [15], who observed a change in porosity with build height (Z-plane). In their study, the porosity increased from 1.1 to 2.7%, which was reported to be due to an increase of melt pool depth and temperature. In the present study, small changes of porosity were obtained in the part XY placement, but not for the build height. Furthermore, the overall porosity obtained in the printed samples was less than 0.1% vol., which is the requirement for example, for biomedical certified products [8]. The pore size distribution ranged between 17 and 159 $\mu$m as detailed in Table 4, detected using VGDefx module analysis. The number of pores analysed was approximately 5000, almost all of which exhibited a spherical shape.

**Table 3:** Results from the VGDefX analysis on the % defect volume ratio in the tensile gauge section of the as-built Ti-6Al-4 V alloy

Defect volume with part placement		
XY-plane	Z-plane	
	Bottom (%)	Top (%)
Left	0.07	0.09
Middle	0.04	0.04
Right	0.01	0.01

**Table 4:** Results from the VGDefX analysis on the pore sizes ($\mu$m) in the tensile gauge section of the as-built Ti-6Al-4 V alloy. Note the X, Y, Z build plate positions are illustrated in Fig. 1

Pore sizes with part placement		
XY-plane	Z-plane	
	Bottom ($\mu$ m)	Top ($\mu$ m)
Left	17 – 136	17 – 159
Middle	17 – 157	17 – 121
Right	17 – 111	23 – 107

The post-thermal HIP samples exhibited porosity of less than 0.01%, demonstrating the effectiveness of this treatment in the elimination of gas voids, due to their diffusion into the alloy, at the high pressures and temperatures used [21]. As reported by Atkinson et al. [26], the driving force for closure of an isolated spherical pore is expressed in terms of pressure (*p*), such that $\gamma$ is the specific energy (1 J/m^2^) of the internal surface of the pore and the *r* is the radius of the curvature of the pore surface.

2 $$p=\frac{2 \gamma }{r}$$

Using the formula to calculate the pressure needed to eliminate the pores, the driving force for the smallest pore of 17 m$\mu$ in diameter is 0.20 MPa, whilst that required for a pore of 159 $\mu$m is 0.03 MPa [26]. Thus, a higher pressure is required for the removal of small pores, compared with that of larger pores. These pressures are well below that typically used for HIP treatments (< 100 MPa) and thus explains the effectiveness of this treatment for the removal of voids within as-built components [27].

### Microstructure characterisation

**Fig. 3:**
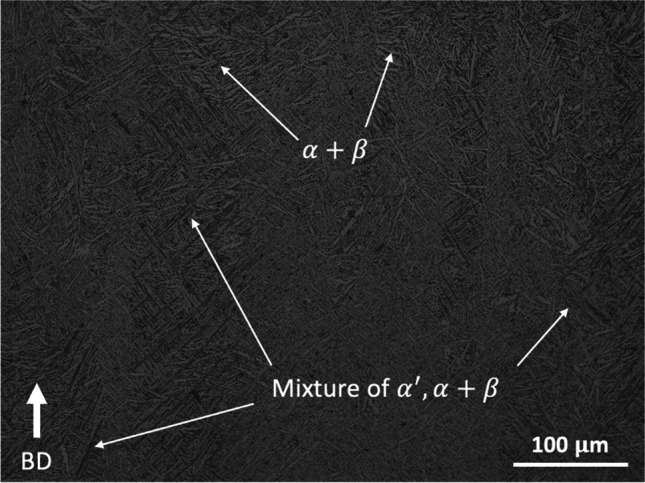
Optical microscope image of as-built titanium alloy highlighting the $\alpha$ laths and prior β grains which grow epitaxially along the build direction (BD)

Examination of the Ti-6Al-4 V alloy parts show Widmanstatten laths alongside with elongated, columnar prior β grains, which grow epitaxially along the build direction (BD), as illustrated in Fig. 3. In addition to this, the microstructure also shows the formation of the needle-shaped martensite $\left(\alpha'\right)$ phase. These grains were measured to be in the range of 75 $\pm$ 13 $\mu$n, 91 $\pm$ 15 $\mu$m, and 75 $\pm$ 15 $\mu$m located 20, 90, and 160 mm in the Z-plane, respectively. This morphology is more widely observed in additively manufactured Ti-6Al-4 V due to the rapid heating and fast cooling rates achieved during the LBF process. Furthermore, the metastable $\alpha'$ microstructures possess a similar crystal structure to that of equilibrium α phase. However, due to rapid solidification, the alloying elements are unable to achieve an equilibrium lattice structure, thus resulting in a distorted hexagonal close pack (*hcp*) crystal structure [11]. The distorted crystal structure of $\alpha'$ can impede the dislocation motion along the crystallographic planes, resulting in high tensile strength but causes premature failure and low ductility [28]. Conversely, Tan et al. [29] highlighted that the introduction of some amount of $\alpha$ martensites by means of parameter optimisation is a promising approach to yield a good combination of high strength and enhanced ductility in AM Ti-6Al-4 V alloy.

**Fig. 4:**
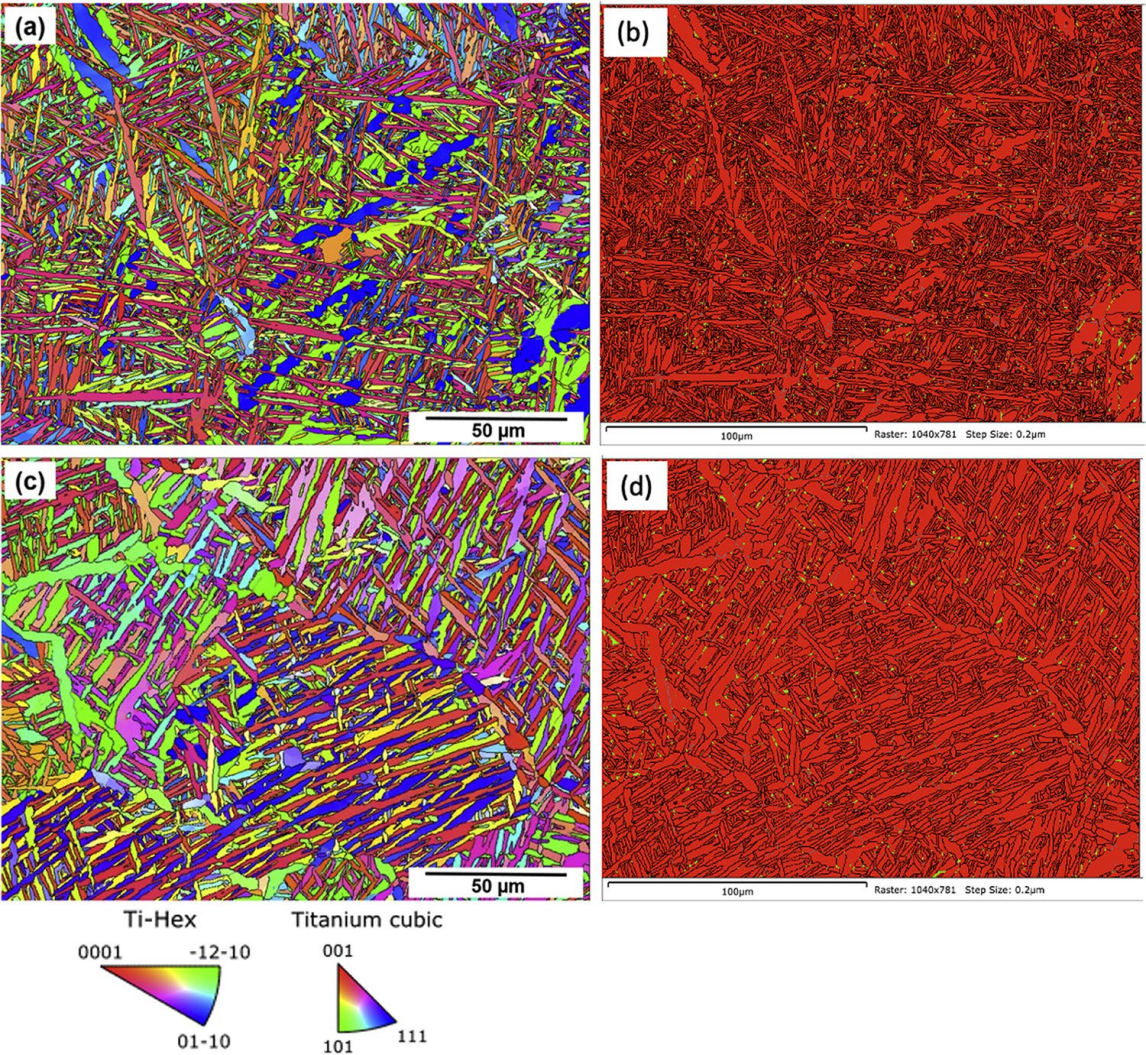
EBSD Inverse Pole Figure (IPF) (**a, c**) and α/β phase distribution (**b, d**) of as-built (**a, b**), and HIPed (**c, d**) Ti-6Al-4 V samples. The red colour (**a, d**) denotes the *α* phase and the green tinge indicates the *β* phase distribution

The grain orientation of the Ti-6Al-4 V samples were analysed using EBSD Inverse Pole Figure (IPF) as shown in Fig. 4. The *hcp α* phase texture is represented by (0001), (–12–10), and (01–10) direct pole figures. Very weak texture can be observed, and no changes in the preferential growth orientation appear in the as-built condition and after the HIP process. However, due to the high thermal input received from the HIP process, the HIPed samples (Fig. 4c exhibits a significant level of grain growth compared to as-built samples (Fig. 4a). This increase in lath morphology is typically observed for isothermally treated components close to the *β* transus temperature of the alloy at 995 °C [11]. The above findings also highlight the effect of temperature on the laths growth and the average lath width in the case of HIPed Ti-6Al-4 V samples. By post-processing the EBSD scanned datasets, the proportion of *α* and *β* phases in the as-built and HIPed samples, were measured as shown in Fig. 4b, d, respectively. The micrograph shows that the samples are dominated by a significant proportion of *α* phase (in red colour) with a negligible amount of *β* phase (in green colour). Based on the quantitative measurement of *α/β* phase distribution shown in Table 5, the area fraction of the *β* phase content in the as-built components with respect to build height did not have a significant change. However, an increase of 25% *β* phase content can be observed after HIP treatment.

**Table 5:** *α/β* phase distribution in the as-built and HIP condition Ti-6Al-4 V with respect to the z-bottom and z-top

	As-built		HIPed treated	
	α phase (%)	β phase (%)	α phase (%)	β phase (%)
Bottom	99.20 $\pm$ 0.20	0.80 $\pm$ 0.20	98.97 $\pm$ 0.45	1.03 $\pm$ 0.45
Top	99.20 $\pm$ 0.26	0.80 $\pm$ 0.20	99.00 $\pm$ 0.26	1.00 $\pm$ 0.26

Table 6 details the size distribution of the $\alpha$ lath width across the entire build area. Taking the left XY-plane for example, the $\alpha$ lath widths located at 20, 90, and 160 mm build heights were 1.00 $\pm$ 0.28 $\mu$m, 0.96 $\pm$ 0.28 $\mu$m, and 0.96 $\pm$ 0.26 $\mu$m, respectively. Consequently, the size distribution of the $\alpha$ lath after the treatment, increased nearly two-fold.

**Table 6:** Distribution of $\alpha$ lath widths obtained from the as-built and HIP condition across the XY-plane with respect to the Z-plane

	α lath widths with part placement			
Condition	XY-plane	Z-plane		
		Bottom ($\mu$ m)	Middle ($\mu$ m)	Top ($\mu$ m)
As-built	Left	1.00 $\pm$ 0.28	0.96 $\pm$ 0.28	0.96 $\pm$ 0.26
	Middle	0.98 $\pm$ 0.30	0.90 $\pm$ 0.27	0.96 $\pm$ 0.30
	Right	1.03 $\pm$ 0.03	0.91 $\pm$ 0.07	0.94 $\pm$ 0.07
HIP	Left	1.69 $\pm$ 0.46	1.72 $\pm$ 0.60	1.75 $\pm$ 0.33
	Middle	1.73 $\pm$ 0.36	1.60 $\pm$ 0.37	1.56 $\pm$ 0.32
	Right	1.50 $\pm$ 0.42	1.70 $\pm$ 0.46	1.49 $\pm$ 0.37

### Phase analysis

**Fig. 5:**
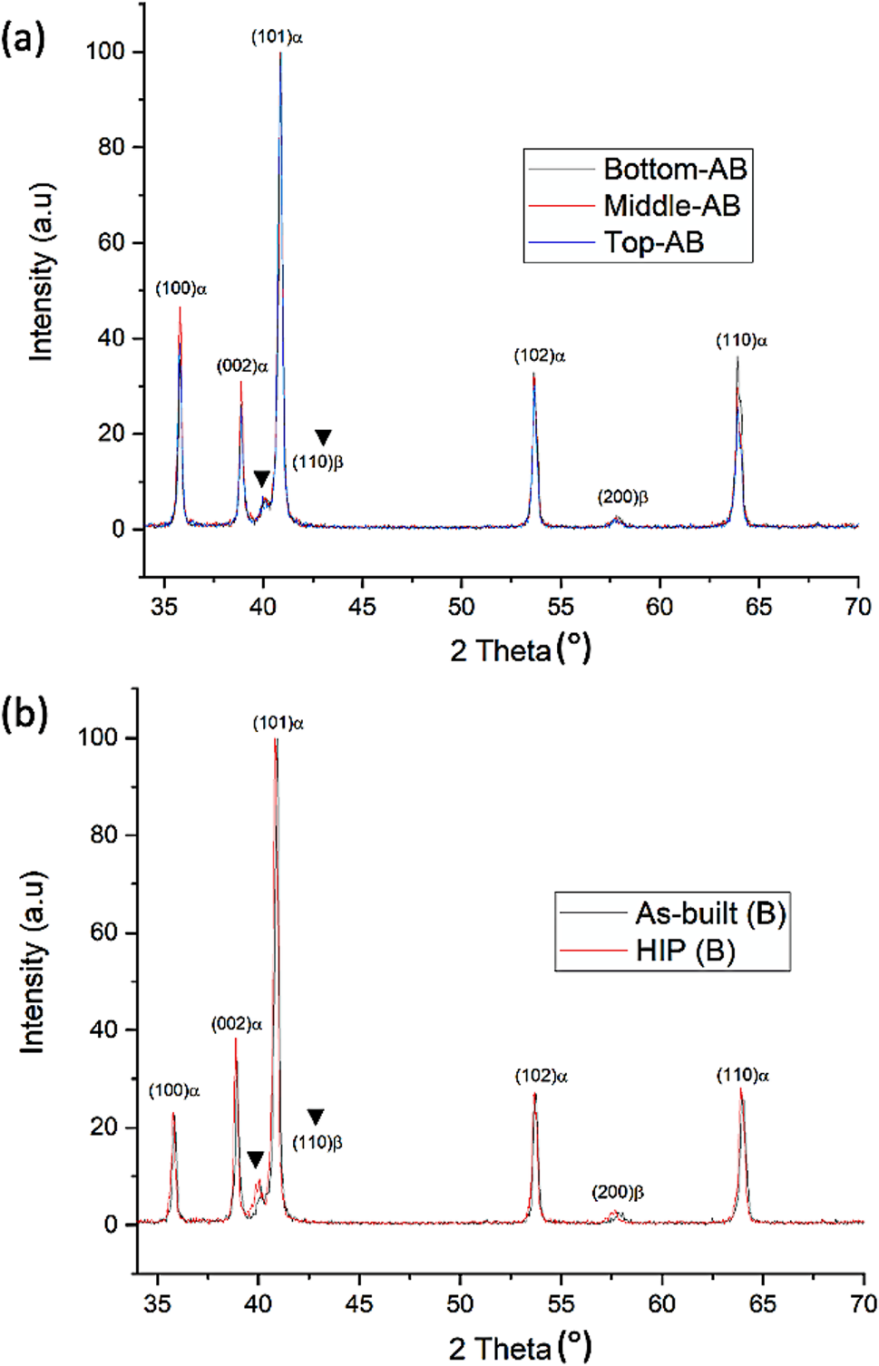
XRD pattern of Ti-6Al-4 V alloy in the as-built condition across the Z-plane (**a**) and after HIP treatment taking the z-bottom sample as an example (**b**)

Figure 5A, b illustrates the XRD patterns of the as-built and HIP-treated components obtained at the bottom, middle, and top positions, across the 170 mm obtained in the Z-plane. The phase composition identified in these spectra is very similar and consisted mainly of $\alpha$ phase peaks and a relatively small fraction of $\beta$ phase peaks, at a lower angle ${(110)}_{\beta }$ and higher angle ${(200)}_{\beta }$ plane reflection. Since the equilibrium $\alpha$ and metastable ${\alpha }^{^{\prime}}$ martensites have the same *hcp* crystal structure, it is difficult to assign the peaks with certainty. However, the presence of two $\beta$ phase peaks gives a strong indication that the phase composition of the as-built components is a mixture of equilibrium $\alpha +\beta$ phases that co-exist with ${\alpha }^{^{\prime}}$ martensites. The lattice parameters for $\alpha$*-hcp* (*a* and *c*) and $\beta$*-bcc* (*a*) were calculated using the *d-spacing* corresponding to ${(100)}_{\alpha }$*,* ${(002)}_{\alpha }$ and ${(110)}_{\beta }$ planes, following Braggs Law (Table 7). The $\alpha$*-hcp* and $\beta$*-bcc* lattice parameters for the as-built components across the build area do not vary significantly. The $c/a$ ratio was calculated to be 1.599 Å, and the *a* lattice of the $\beta$*-bcc* ranges between 3.172 Å and 3.175 Å. Xu et al. [30] reported that in-situ decomposition of ${\alpha }^{^{\prime}}$ martensites to equilibrium $\alpha +\beta$ phases exhibits $\beta$*-bcc a* lattice parameter between 3.18 and 3.21 Å and $c/a$ ratio of $\alpha$*-hcp* close to 1.590–1.600 Å. These values can be used as a benchmark to predict whether significant martensite decomposition occurred during the PBF-LB processing. The obtained lattice parameters and $c/a$ ratio are within the reported range [30], thus providing further evidence that the phases present in the as-built condition were near equilibrium $\alpha +\beta$ lamellar and that partial in-situ decomposition of ${\alpha }^{^{\prime}}$ martensites occurred.

**Table 7:** Lattice parameters ($\alpha$-Ti and $\beta$-Ti), $c/a$ ratio, and $\beta$ phase ratio in the as-built and HIP condition Ti-6Al-4 V with respect to the z-bottom, z-middle and z-top

Condition	Z-plane	a-lattice $\alpha$-Ti (Å)	c-lattice $\alpha$-Ti (Å)	$c/a$ ratio (Å)	a-lattice $\beta$-Ti (Å)	$\beta$ phase ratio (%)
As-built	Bottom	2.894 $\pm$ 0.004	4.627 $\pm$ 0.007	1.599	3.172 $\pm$ 0.010	6.8 $\pm$ 0.3
	Middle	2.897 $\pm$ 0.006	4.631 $\pm$ 0.008	1.599	3.174 $\pm$ 0.007	6.9 $\pm$ 0.6
	Top	2.897 $\pm$ 0.002	4.630 $\pm$ 0.002	1.598	3.175 $\pm$ 0.006	6.2 $\pm$ 0.7
HIP-treated	Bottom	2.890 $\pm$ 0.006	4.621 $\pm$ 0.009	1.599	3.176 $\pm$ 0.006	8.0 $\pm$ 1.5
	Middle	2.892 $\pm$ 0.004	4.622 $\pm$ 0.006	1.599	3.180 $\pm$ 0.007	8.1 $\pm$ 1.4
	Top	2.892 $\pm$ 0.002	4.623 $\pm$ 0.003	1.599	3.180 $\pm$ 0.006	8.3 $\pm$ 1.2
Xu et al. [30]	$\alpha +\beta$			1.590 – 1.600	3.18 – 3.21	

In order to obtain an indication of the $\beta$ phase content in the samples by XRD, a comparison was made between the relative intensities of the ${(101)}_{\alpha }$ peak and the ${(110)}_{\beta }$ phase peaks both before and after HIP treatment [11]. The $\beta$ ratio percent was determined to be an average of 6.6%, which was consistent across the build area. Comparing the XRD pattern of samples before and after HIP treatment in Fig. 5, it is noticeable that there is a significant increase of the ${(110)}_{\beta }$ peak relative to ${(101)}_{\alpha }$, with a β ratio increase to ~ 8.1%. Lu et al. [31] and Yan et al. [32] reported a well-defined ${(110)}_{\beta }$ peak indicates the increase presence of *β* phase in the material. Overall, based on the method of relative peak intensities [11], the $\beta$ ratio percent of the samples after HIP treatment increased by 23%. This result is consistent with the obtained EBSD evaluation, in which the *β* phase distribution increased by 25% after HIP treatment. This indicates that the use of the XRD peak relative intensity ratio provides a measure of the relative changes in $\beta$ content, when EBSD analysis results are not available.

In addition, associated with the HIP treatment was a slight expansion of the $\beta$ phase crystal structure as shown in the $\beta$*-bcc* lattice values listed in Table 7. Elmer et al. [33] reported that the lattice expansion of the $\beta$ phase is strongly affected by the combination of thermal and chemical effects during the $\alpha \to \beta$ phase transformation. Thampy et al. [34] correlated the shift of $\beta$*-bcc* lattice parameter to larger values with slower cooling rates due to the HIP treatment.

### Microstrain analysis

Gaussian–Lorentzian curve fitting was used to acquire the full-width half maximum (FWHM) using the following 6 diffraction intensity peaks (${(100)}_{\alpha }$, ${(002)}_{\alpha }$, ${(101)}_{\alpha }$, ${(102)}_{\alpha }$, ${(110)}_{\alpha }$, and ${(103)}_{\alpha }$). The Williamson-Hall method [24] was applied to examine the lattice strain of the samples across the build area. Table 8 illustrates the microstrain distribution with build height for the as-built and HIP-treated alloy samples. Based on the results, no notable difference in microstrain can be observed with part print location for either set of samples. This is confirmed using a single factor ANOVA test with a null hypothesis stating that the variances with build conditions are equal. The resulting *p* value was 0.19. As the p value is greater than 0.05 (*p* value > 0.05), it is considered ‘insignificant’ and the null hypothesis has to be accepted [35]. This suggests that the thermal gradient across the powder bed is homogeneous along the XYZ-direction during manufacturing. There is however a reduction in stress between the as-built and HIP-treated samples. This is observed through the decrease in average micro-strain of 0.006 to 0.004 for the as-built and HIP condition, respectively. Given that the micro-strain slightly decreases, it is expected that mechanical properties will increase [36]. However, as HIP treatment is carried out above the stress relaxation temperature of ~ 400 °C [11], martensitic decomposition occurs which results in increased *β* content and lath coarsening, resulting in decreased tensile strength. According to ANOVA analysis, the micro-strain after HIP treatment also showed no significant difference with height (*p* value > 0.05 [35]).

**Table 8:** Microstrain distribution within the crystal lattice across the build area of as-built and HIP condition Ti-6Al-4 V with respect to the z-bottom, z-middle, and z-top

Condition	Z-plane	Micro-strain
As-built	Bottom	0.006 $\pm$ 0.0009
	Middle	0.006 $\pm$ 0.0005
	Top	0.005 $\pm$ 0.001
HIP-treated	Bottom	0.004 $\pm$ 0.0009
	Middle	0.004 $\pm$ 0.001
	Top	0.005 $\pm$ 0.0007

### Mechanical investigation

Low-force hardness measurements were carried out to determine the degree of plastic deformation. The Vickers hardness HV0.5 results are presented in Table 9. For example, samples located on the left, z-20 mm and z-160 mm, vary from 345 $\pm$ 4 to 346 $\pm$ 11, respectively. This shows that there was no systematic difference across the build area as confirmed using ANOVA analysis (*p* value > 0.05 [35]). The HIP samples, however, exhibited a significant decreased in hardness, demonstrating the softer nature of the alloy after this thermal treatment. Eshawish et al. [37] also reported lower micro-hardness for HIP Ti-6Al-4 V alloy and improved fatigue properties.

**Table 9:** Average Vicker’s hardness (HV 0.5) of as-built and HIP condition Ti-6Al-4 V across the XY-plane with respect to the Z-plane

	Hardness measurement with part placement			
Condition	XY-plane	Z-plane		
		Bottom ($HV0.5$)	Middle ($HV 0.5$)	Top ($HV0.5$)
As-built	Left	345 $\pm$ 4	348 $\pm$ 5	346 $\pm$ 11
	Middle	343 $\pm$ 6	336 $\pm$ 6	341 $\pm$ 3
	Right	341 $\pm$ 6	344 $\pm$ 2	346 $\pm$ 4
HIP	Left	325 $\pm$ 7	326 $\pm$ 9	325 $\pm$ 10
	Middle	339 $\pm$ 11	328 $\pm$ 12	330 $\pm$ 11
	Right	335 $\pm$ 14	322 $\pm$ 6	320 $\pm$ 6

**Fig. 6:**
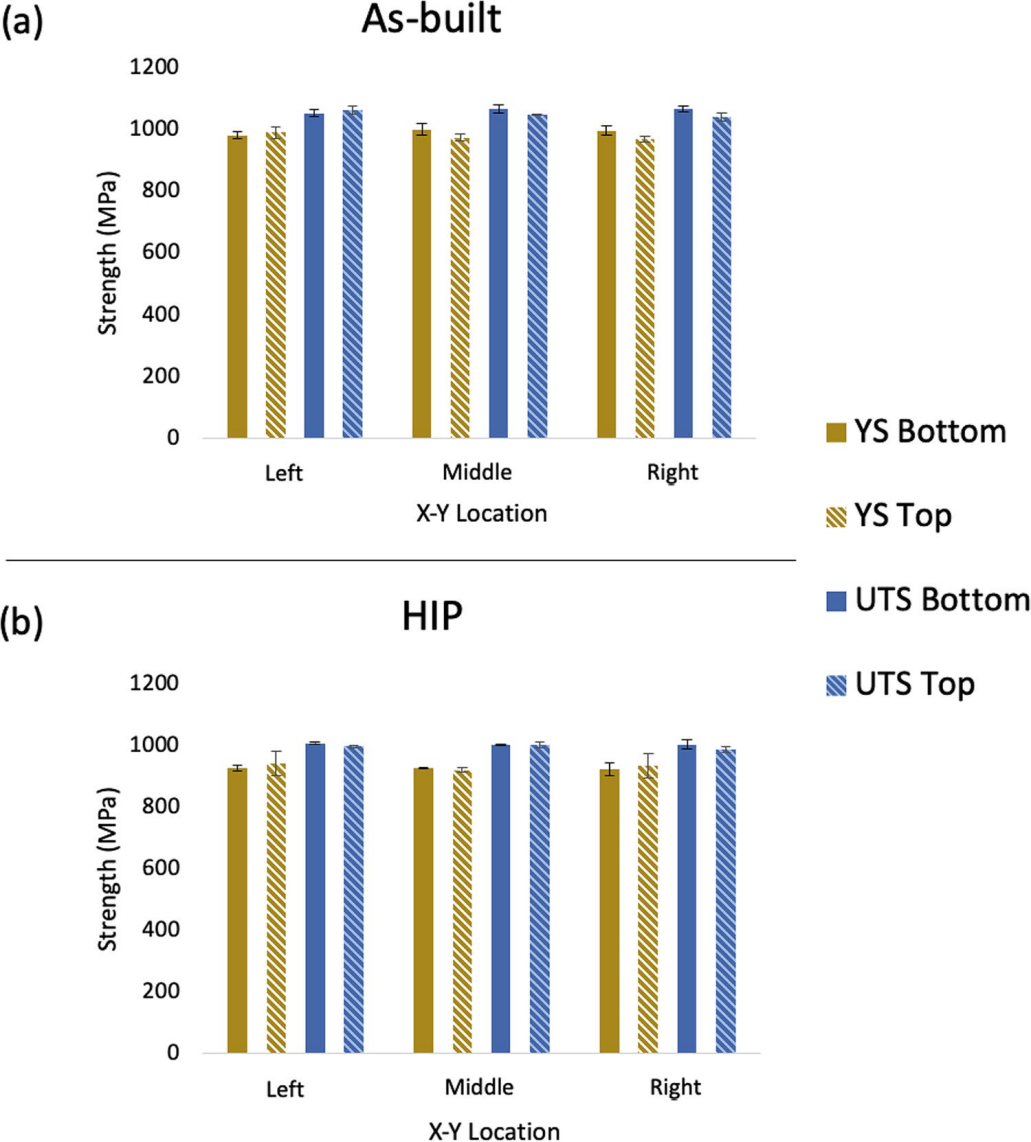
Mechanical properties of as-built (**a**) and HIP (**b**) tensile specimens showing the influence of Z-plane; bottom and top with respect to the XY-plane location on the yield strength (YS) and ultimate tensile strength (UTS)

Figure 6 shows the variation in tensile properties before and after the HIP treatment. As detailed earlier, the tensile tests were obtained by machining from cylindrical rods presented in Fig. 1. The as-built components demonstrated a high mechanical performance of 1000 $\pm$ 20 MPa YS, 1068 $\pm$ 13 MPa UTS, and 14 $\pm$ 2% elongation. The variation of tensile properties was analysed using ANOVA in terms of build location (0.70–1.0, *p* value) and height (0.2–0.4, *p* value), and the results showed no significant differences as the values are greater than 0.05 (*p* value > 0.05 [35]). Plastic formability is generally associated with the number of slip systems in the alloy [38]. The slip system of $\beta$*-bcc* is higher (48 slip systems), compared with that of $\alpha$-*hcp* (3 slip systems). Therefore, the $\beta$*-bcc* phase is soft and ductile, relative to the much stronger $\alpha$-*hcp* [38]. There are several regions of decomposed needle-like laths as shown in the optical micrographs (Fig. 3). These fine laths act as a strengthening feature, increasing the number of grain boundaries, impeding dislocation motion along the crystallographic planes. Additionally, the higher $\alpha$ phase fraction (~ 90%), relative to the $\beta$ phase calculated through the XRD graphs, suggests that the stronger $\alpha$-*hcp* phase is dominant, thus leading to superior mechanical strength. It is also interesting to note that despite the difference in porosity content of the as-built condition across the XY-plane, the ductility was consistent, signifying the insignificant effect of porosity at the low levels found.

The alloy’s properties changed slightly after HIP treatment. For example, the highest measurement recorded was 940 $\pm$ 40 MPa YS, 1007 $\pm$ 3 MPa UTS, and 17 $\pm$ 0.5% elongation. The associated 7% and 6% decrease in YS and UTS, respectively, is associated with a coarsening of $\alpha$ laths, based on the classic Hall–Petch relation expressed in Eq. 3 [39].

3 $${\sigma }_{\in }={\sigma }_{{0}_{\in }}+k/\sqrt{l}$$

The yield stress, ${\sigma }_{\in }$, is expressed in terms of the average resolve stress of the material (${\sigma }_{{0}_{\in }}$), the Hall–Petch constant (*k*), and the microstructure feature ($l$), which in this case is the $\alpha$ laths. The Hall–Petch relationship shows that the yield stress is inversely proportional to the square root of the $\alpha$ lath widths thereby illustrating the microstructure-strength dependency. The 17% increase of elongation, as illustrated Table 10, can be accounted for through the increase in $\beta$ phase. Similarly, Leuders et al. [16] reported that the increased sample ductility occurs due to the increased amount of $\beta$*-bcc* phase caused by high temperature treatments. This result is also reflected in the obtained hardness measurements. The slight stress relieving of HIP treatment (Table 8) did not appear to cause any significant changes in the mechanical performance of the alloy. This demonstrates that the in-situ stress relieving during printing was sufficient to minimise the effect of stress in the part properties.

**Table 10:** Average % elongation of as-built and HIP condition Ti-6Al-4 V across the XY-plane with respect to the Z-plane

	% elongation with part placement		
Condition	XY-plane	Z-plane	
		Bottom (%$El$)	Top (%$El$)
As-built	Left	13.4 $\pm$ 1.4	14.9 $\pm$ 1.0
	Middle	14.0 $\pm$ 2.0	14.6 $\pm$ 1.6
	Right	14.0 $\pm$ 1.7	14.15 $\pm$ 1.5
HIP	Left	15.6 $\pm$ 1.4	16.6 $\pm$ 2.1
	Middle	16.2 $\pm$ 11	16.1 $\pm$ 1.3
	Right	16.8 $\pm$ 0.5	16.5 $\pm$ 1.0

It should be noted that although there was a change in mechanical performance after HIP treatment, the as-built condition still exceeded the recommended minimum mechanical properties set for the Ti-6Al-4 V alloy for use, for example, in a biomedical implant (795 MPa YS and 860 MPa TS, 10% Elongation). This highlights the potential for high volume printing of parts using the laser system, potentially avoiding the need for additional post-processing methods, thus significantly reducing the cost and time involved.

### Fractography analysis

**Fig. 7:**
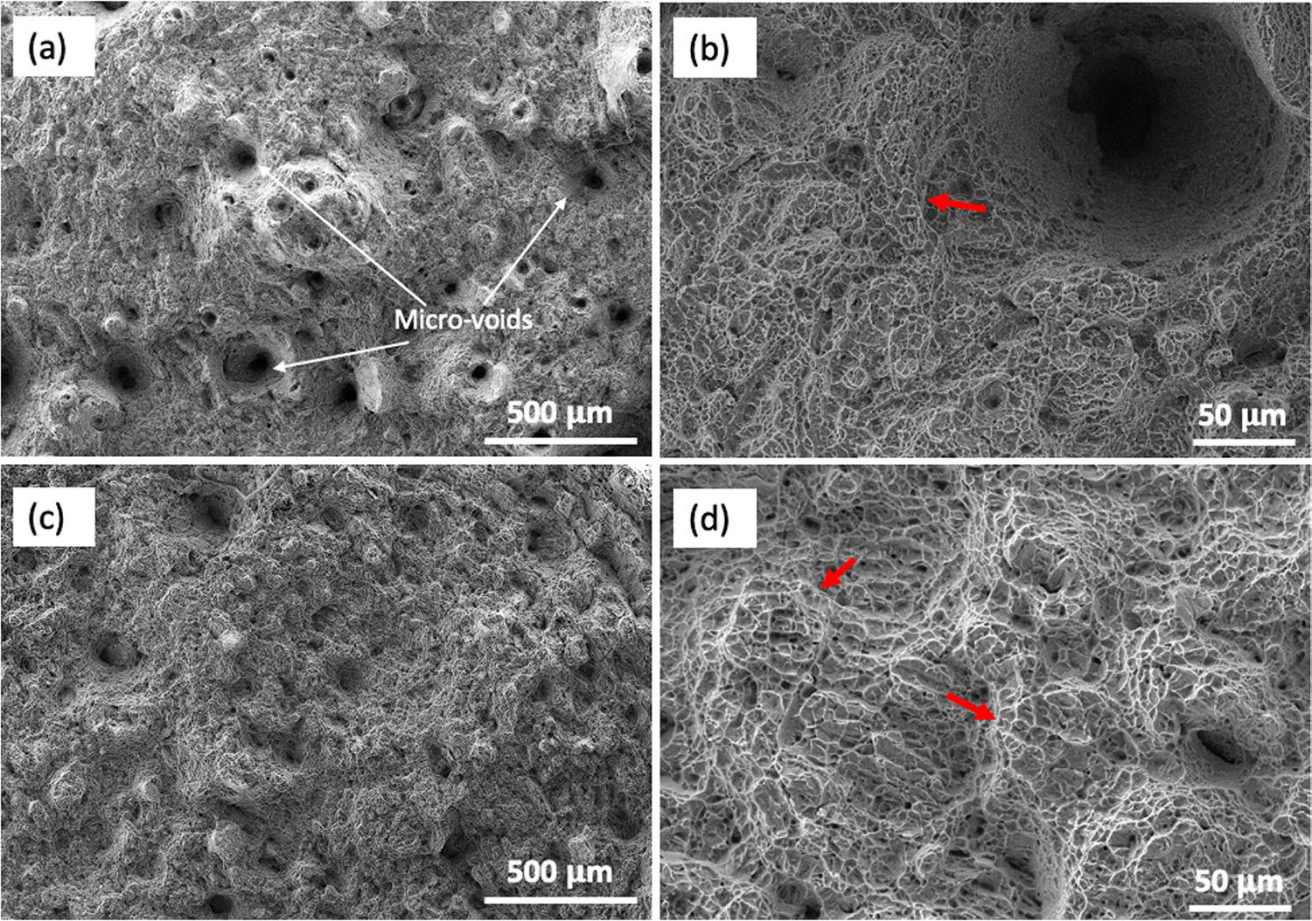
SEM micrographs of the fracture surfaces of as-built (**a, b**) and HIP (**c, d**) conditions illustrating the presence of micro-voids, dimples, and crack path propagation (as highlighted in red arrows)

To obtain more information as to why there are differences in the tensile behaviour between the as-built and after HIP treatment, fractography was carried out on the fracture surfaces of the broken tensile specimens as shown in Fig. 7. A noticeable number of micro-voids are observed in the as-built fracture surface (Fig. 7a), when compared to that of the HIP samples (Fig. 7c). It has been widely reported that pores are detrimental for fatigue performance, especially pores that are located around the surface and sub-surfaces causing high concentration initiation sites for crack propagation to occur [16]. However, pores below the critical size of 450–500 $\mu$m [40, 41] and less than 1% vol. defect [17], typically, do not affect the tensile strength of the material. In this study, the as-built samples exhibited low porosity (< 0.09% vol. defect) with pore sizes (17–159 $\mu$m) in the range below the critical size. Above the critical size, pores will be dominant, significantly affecting the mechanical performance and possibly resulting to premature failure of parts. Therefore, it appears as though the properties of PBF-LB TI-6Al-4 V are closely associated by the changes in microstructure ($\alpha$ laths and $\beta$ phases), not the porosity content, over the range investigated. Upon closer inspection of the fracture, Fig. 7b, d shows ‘dimple’ regions, indicating ductile fracture [42]. However, when comparing the size of dimples between the two conditions, HIP samples exhibit larger dimples, which is likely to be associated with the bigger $\alpha$ laths [43]. There are also some signs that the surfaces predominant fracture is intergranular, whereby the cracks have propagated along the prior *β* grain boundaries as shown by the red arrows. The intergranular fracture is likely caused by the high anisotropic property of the columnar prior* β* grain having a preferred crystallographic orientation at (110) plane [44].

## Discussion

### Porosity–spatter direction

**Fig. 8:**
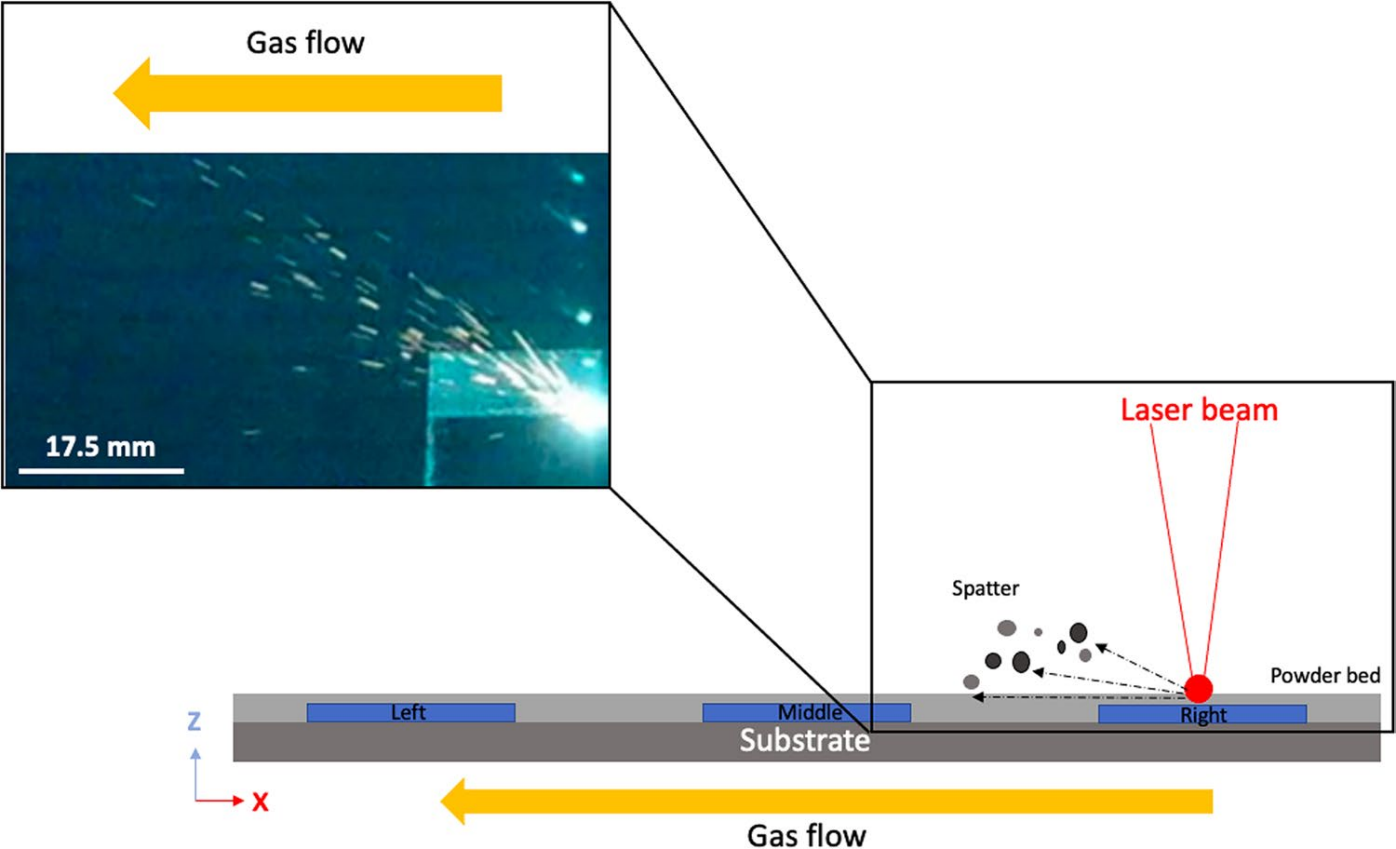
Schematic diagram of the PBF-LB build platform illustrating the gas flow direction and the spattering of particles across the XY-plane (adapted from [48])

As reported by Cunningham et al. [45], spherical gas voids are unavoidable defects and form through pore transfer from the feedstock powder particles or inert gas precipitation trapped within the part during the melting process [46]. Further, Gong et al. [17] reported that pores can also occur due to the splashing of the molten metal during printing and when redeposited, resulting in the formation of thin discontinuous tracks, which in turn can influence the formation of spherical defects within the part. Wang et al. [47] described the splashing of molten material as ‘spatter’ and considered it into three categories; 1. droplet spatters coming from the melt-pool surface instability, 2. spatters included in the metallic jet and coming from the recoil pressure zone, and 3. non-melted powder spatters at the front of the melt-pool. However, Keaveney et al. [48] investigated the spatter generation using in-situ optical emission monitoring system using the same model of the PBF-LB Renishaw system. It was demonstrated that the spatter particles were projected towards the left side of the build area by the flow of argon gas, regardless of the melt pool flow (Fig. 8). Subsequently, these spattered particles can spread further during powder recoating. Based on these results, it appears that printing closer to the Ar gas outlet can significantly reduce the accumulation of spattered powder and in turn decreases the amount of porosity within the printed part [49]. Ladewig et al. [50] reported that optimisation of gas flow rate such that it should be high as possible can help facilitate the removal of process by-products and to avoid redeposits. In addition to the influence of the direction of gas flow, melt splashing occurs due to the melt pool instability which is responsible for increased porosity in the material [51]. Based on the literature and results presented in this study, a likely cause of the increased porosity in the XY-plane (from right to left), is due to increased levels of melt pool spatter. These particles are carried by the flow of argon gas and deposited at higher concentrations further from the source.

### Micro-structure homogeneity

The study found that $\alpha$ lath width, $c/a$ ratio, and $\beta$ phase fraction were homogenous across the build area. This finding differs from the results obtained by Lui et al. [7], who reported microstructural gradient across the Z-plane of PBF-LB Ti-6Al-4 V components. The changes in microstructure such as $\alpha$ lath width (${\lambda }_{2}$) are typically associated with cooling rate ($\dot{T}$) as illustrated in Eq. 4, where *B* and *n* are the materials constant [52]. As the findings of this study indicate homogenous microstructure, this suggests that the cooling conditions (solidification and phase transformation) during printing were consistent and significantly homogenous across the build area.

4 $${\lambda }_{2}= \frac{B}{{\dot{T}}^{n}}$$

A possible explanation for the microstructure homogeneity observed in this study is that the sample clusters in the large volume printing promoted retention of high thermal mass (*C*_th_). The ability of an object to store thermal energy is correlated to heat capacity in unit JK^−1^. For an object with a uniform composition such as Ti-6Al-4 V alloy in this case, *C*_th_ can be estimated using Eq. 5 [53].

5 $${C}_{th}={mc}_{p}$$

where *m* is the mass of the object and *C*_*p*_ is the heat capacity of the material at a constant pressure*.* Although the sample clusters in the XY-plane shown in Fig. 1 were printed approximately 60 mm away from one another, the four individual parts in the sample clusters are close enough to act as an insulator adjacent to one another. This result is comparable to that of Tan et al. [29], who carried out a study on build thickness dependent microstructure on Ti-6Al-4 V. It was observed that the thicker samples (20 mm) exhibited larger $\alpha$ laths (0.813 $\pm$ 0.185 $\mu$m), compared to the thinner samples (1 mm) (0.283 $\pm$ 0.068 $\mu$m), indicating that the thicker samples experienced slower cooling rate due to the higher thermal mass. In this study, the size of the sample clusters was approximately 30 mm. This sample size may have helped to effectively retain heat, reducing the thermal gradient, and maintaining a constant cooling rate throughout the entire build area.

## Conclusion

This study investigated for the first time, the variability of Ti-6Al-4 V microstructure and mechanical performance across a large build area of 250 × 250 × 170 mm^3^, in a production scale PBF-LB system. The effect of post-thermal HIP treatment on the alloy samples obtained from different locations over this large area was also examined. The following conclusions were drawn from this study:

1. Increased porosity (0.01 to 0.09%), for samples printed further away from the Ar gas outlet (XY-plane), is likely to be associated with the increased incorporation of condensate and / or spatter particles. The spattered particles generated during powder melting are redeposited, causing discontinuous line tracks, which may result in the formation of spherical voids.
2. No significant difference was observed in the microstructural features (prior* β* grain, $\alpha$ lath thickness and *β* phase fraction) and micro-strain of the as-built condition, across the XY- and Z-plane. This is likely to be facilitated, due to the high thermal mass of the ~ 30 mm sample clusters used, which would help to provide a relatively uniform thermal treatment across the build, thus facilitating microstructural homogeneity.
3. Partial in-situ decomposition of ${\alpha }^{^{\prime}}\to \alpha +\beta$ phases was determined through the lattice constants of $\alpha$*-hcp* and $\beta$*-bcc*, indicating that annealing/stress relief occurred during manufacturing due to the high thermal mass.
4. Mechanical test results obtained from samples tested across the entire build demonstrated that there was no significant variation across the XY- and Z-planes.
5. Samples which were HIP-treated demonstrated pore closure to below 0.01%, a slight reduction in internal residual stresses, $\alpha$ lath coarsening, and higher *β* phase fraction. Due to the relatively low porosity in the as-built samples, the changes observed in the alloy’s mechanical properties appeared to be more closely linked to changes in microstructure, than to pore closure following treatment.

This study demonstrates the ability of a production scale PBF-LB system for the large area printing of Ti-6Al-4 V alloy parts. Printed parts exhibited both a homogenous microstructure and mechanical properties across the 250 × 250 × 170 mm build area. In addition, the as-built Ti-6Al-4 V properties complied with the minimum required strengths for use in the biomedical application (795 MPa YS, 860 MPa TS, 10% Elongation), illustrating that under certain process conditions, post-processing may not be required. However, HIP process still shows enhancement in ductility and a reduction in porosity.
